# Engineering of *Leuconostoc citreum* for Efficient Bioconversion of Soy Isoflavone Glycosides to Their Aglycone Forms

**DOI:** 10.3390/ijms23179568

**Published:** 2022-08-24

**Authors:** Jaewoo Son, Ki Jun Jeong

**Affiliations:** 1 Department of Chemical and Biomolecular Engineering, BK21 Plus Program, KAIST, 291 Daehak-ro, Yuseong-gu, Daejeon 34141, Korea; 2 Institute for The BioCentury, KAIST, 291 Daehak-ro, Yuseong-gu, Daejeon 34141, Korea

**Keywords:** lactic acid bacteria, *Leuconostoc citreum*, soy isoflavone, bicistronic design, bioconversion

## Abstract

Soy isoflavones are phytochemicals that possess various beneficial physiological properties such as anti-aging, anti-tumor, and antioxidant properties. Since soy isoflavones exist in glycoside forms, their bioavailability requires initial hydrolysis of the sugar moieties bound to them to be efficiently absorbed through the gut epithelium. Instead of conventional chemical hydrolysis using acids or organic solvents, alternative strategies for enhancing the bioavailability of soy isoflavones using biological methods are gaining attention. Here, we engineered *Leuconostoc citreum* isolated from Korean kimchi for efficient bioconversion of soy isoflavone glycosides into their aglycone forms to enhance their bioavailability. We first constructed an expression module based on the isoflavone hydrolase (IH)-encoding gene of *Bifidobacterium lactis*, which mediates conversion of isoflavone glycosides to aglycone forms. Using a high copy number plasmid and bicistronic expression design, the IH was successfully synthesized in *L. citreum*. Additionally, we determined enzymatic activity of the IH using an in vivo β-glucosidase assay and confirmed its highly efficient bioconversion efficiency for various types of isoflavone glycosides. Finally, we successfully demonstrated that the engineered *L. citreum* could convert isoflavone glycosides present in fermented soymilk into aglycones.

## 1. Introduction

Edible plants are dietary sources of numerous phytochemicals, which are typically secondary metabolites that occur in plant-derived foods. Among the various classes of phytochemicals, polyphenols, which include phenolic acids, flavonoids, and stilbenes are the major classes of bioactive phytochemicals [1]. One of the important physiological properties of these molecules is their superior ability to promote human health and reduce oxidative stress in mammals [1]. Soy isoflavones present in various plants, particularly in soybeans, are classified as flavonoids with a structure similar to estrogen and possess both estrogen-agonist and estrogen-antagonist properties [1,2]. In recent years, the potential health benefits of soy isoflavones have been revealed to include numerous physiological properties such as anti-aging and anti-tumor properties [2,3]. Furthermore, soy isoflavones have the ability to improve learning and memory and also aid in preventing heart diseases, diabetes, and Kawasaki disease [2]. Isoflavones also possess protective antioxidant properties that help prevent oxidative stress by reducing the generation of free radicals and reactive oxygen species (ROS) via decomposition of hydrogen peroxide and quenching of singlet oxygen [1,4]. In their natural environment, soy isoflavones exist in glycoside forms (genistin, daidzin, and glycitin) which bind to sugar molecules. However, to be absorbed in mammals, the bound sugar molecules need to be released, thus leading to the formation of aglycones such as genistein, daidzein, and glycitein [5]. In soybeans, since most isoflavones exist in glycoside forms, their bioavailability requires initial hydrolysis of the sugar moieties by isoflavone hydrolases produced by commensal bacteria present in the gut, because mammals do not possess intrinsic isoflavone hydrolases [6,7]. In this regard, to enhance the bioactivity and bioavailability of soy isoflavones, various strategies based on chemical methods, including hydrolysis of isoflavone glycosides into aglycone forms using acid or organic solvents, have been developed for the extraction of isoflavones [8,9,10]. However, these methods are energy-intensive, and byproducts produced during the extraction can result in environmental pollution and safety issues for downstream applications. Consequently, alternative strategies that use biological methods relying on microorganisms or immobilized enzyme systems to enhance the bioavailability of soy isoflavones are gaining attention [11,12].

Lactic acid bacteria (LAB) are a well-known group of non-sporulating gram-positive bacteria composed of several different genera, including *Lactococcus*, *Lactobacillus*, *Leuconostoc*, *Pediococcus*, *Streptococcus*, and *Oenococcus* [13]. Historically, they have long been used in the manufacture of fermented foods such as milk and vegetables to enhance taste and natural preservation [14]. In recent years, LAB are drawing increasing attention for novel purposes, owing to their safe use for human and animal consumption, metabolic versatility, and a wide ecological niche [15]. Furthermore, beneficial properties such as simple carbon metabolism, effective energy consumption, and a small genome make LAB crucial candidates for metabolic engineering strategies that are focused on regulating synthetic pathways to produce important fermentation end-products including sweeteners, flavors, and vitamins [16]. Considering these beneficial characteristics, LAB have been used along with *Bifidobacterium* as an excellent probiotic for bioconversion of soy isoflavone glycosides [17,18]. Nevertheless, the constraints of anaerobic conditions required for their growth and low conversion efficiency limit their utilization for enhancing the bioavailability of soy isoflavones.

*Leuconostoc citreum* is a hetero-fermentative LAB that plays a significant role in the fermentation of dairy products such as kimchi, milk, vegetables, and meat [19]. Recently, *L. citreum* has been identified as a crucial probiotic candidate to produce vitamins and bioactive antimicrobial peptides, as well as to regulate immune responses [20,21,22]. Currently, *L. citreum* has been attracting attention for its use in food biotechnology owing to its favorable advantages such as non-production of endotoxins and a good growth rate under both aerobic and anaerobic conditions [23]. In addition, recent advances in genetic engineering tools related to *L. citreum* have enabled its application as a potential probiotic strain [24,25,26].

**Figure 1:**
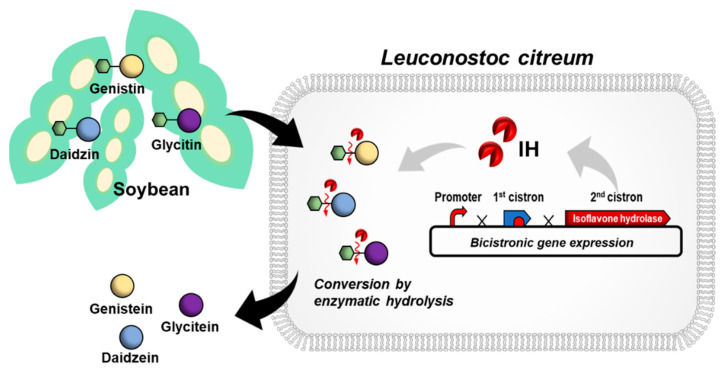
Schematic of *L. citreum* engineering for the efficient bioconversion of soy isoflavone glycoside forms to aglycone forms.

In this study, we report the engineering of *L. citreum* for efficient bioconversion of soybean isoflavone glycosides (Figure 1). β-glucosidase is known as the enzyme that is responsible for the hydrolytic breakdown of carbohydrates such as cellobiose [27], and it can be classified as isoflavone hydrolase with high specificity towards isoflavone glycosidic conjugates. Therefore, an expression module based on the isoflavone hydrolase encoding gene of *Bifidobacterium lactis*, which mediates the conversion of isoflavone glycosides to aglycone forms, was constructed in *L. citreum*. Using a high copy number plasmid (pCB4270) and bicistronic expression design, the isoflavone hydrolase from *B. lactis* (IH) was successfully synthesized in *L. citreum*, and enzymatic activity of the IH was determined using an in vivo β-glucosidase assay. Naturally, there are several types of soy isoflavone glycosides including malonyl, acetyl, and underivatized glucosides, and their content differs depending on product type and processing conditions [2,28]. Among them, the major isoflavones in soybeans are underivatized glucosides such as daidzin, genistin, and glycitin [28,29,30], so we focused on the bioconversion of underivatized glucosides (genistin, daidzin, and glycitin) and confirmed its highly efficient bioconversion efficiency for all those glucosides to their aglycone forms. Finally, using engineered *L. citreum*, we demonstrated that isoflavone glycosides produced during fermentation of soymilk could be converted into aglycones with a high conversion yield.

## 2. Results and Discussion

### 2.1. Expression of the IH Gene in L. citreum for Hydrolysis of Isoflavone Glycosides

To engineer *L. citreum* that is capable of hydrolyzing isoflavone glycosides, it is necessary to develop a glycoside hydrolysis system using a highly specific and efficacious enzyme. Previously, it was reported that LAB, including *Lactobacillus*, *Enterococcus*, *Lactococcus*, and *Bifidobacterium*, have a great potential in hydrolyzing glycosides into aglycones [7]. Youn et al. (2012) isolated a novel isoflavone hydrolase (IH) from *Bifidobacterium lactis* SH5 that showed superior hydrolytic activity against isoflavone glycoside forms [31]. Furthermore, kinetic analysis of this IH revealed that the optimal pH and temperature (30 °C) required for its stability were also suitable for the growth of *L. citreum* [31]. Therefore, we decided to employ IH from *B. lactis* SH5 for efficient production of isoflavone aglycone forms in *L. citreum*.

**Figure 2:**
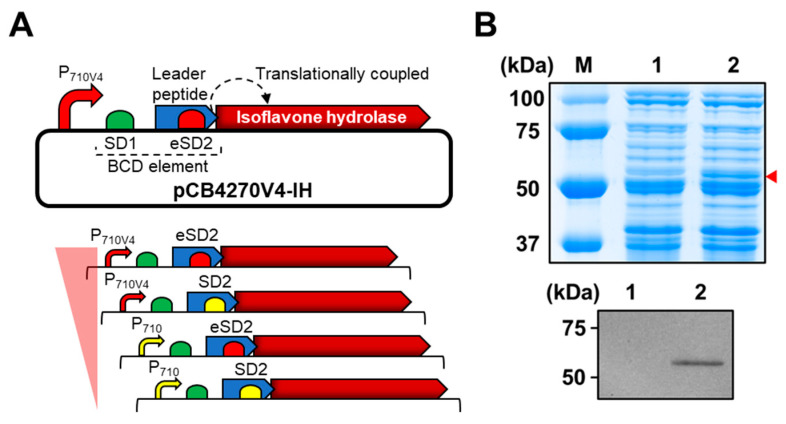
Expression of the gene encoding for IH in *L. citreum* for bioconversion of soy isoflavone glycosides. (**A**) Schematic diagram of bicistronic design (BCD) for expression of the gene encoding for IH. Up, plasmid pCB4270V4-IH constructed for the expression of the gene encoding for IH in *L. citreum*; down, four different expression systems with different strengths of promoters (P_710_ and P_710V4_) and SD (SD2 and eSD2) parts. (**B**) SDS-PAGE and western blot analysis of IH gene expression in *L. citreum* harboring pCB4270V4-IH (P_710V4_ and eSD2). Up, SDS-PAGE analysis. Lane M, molecular weight markers (kDa); Lane 1, soluble fractions of *L. citreum* wild type; Lane 2, soluble fractions of *L. citreum* harboring pCB4270V4-IH. Arrowhead indicates the band of IH; down, confirmation of IH by western blot assay. Lane 1, *L. citreum* wild type; Lane 2, *L. citreum* harboring pCB4270V4-IH.

It is well known that establishing a highly efficient whole-cell biocatalyst is correlated with the content of a particular key enzyme in a cell; therefore, we used the high copy number plasmid pCB4270 [26] to clone the IH-coding gene under the control of bicistronic expression design, which was developed for stable expression of heterologous proteins in *L. citreum* [25] (Figure 2A). In the bicistronic design, the first cistron consisting of the Shine–Dalgarno (SD) sequence 1 (SD1) and a leader peptide (17 amino acids) was positioned between the promoter and IH gene (Figure 2A) to reliably express the target gene in *L. citreum*. As a second SD sequence, SD2 was cloned at the 3′-end of a leader peptide sequence; it serves as a ribosome binding site (RBS) for translation of the IH gene (second cistron) by means of translational coupling (Figure 2A). Four sets of variants with a bicistronic design that enabled the expression of the gene encoding for IH at various intensities based on two different strength promoters (strong P_710V4_ and weak P_710_) and SD sequences (strong eSD2 and weak SD2) were constructed (Figure 2A), and expression level of the IH protein in each variant was analyzed by SDS-PAGE. Among the four sets of variants, we found that the construct, consisting of a strong constitutive promoter (P_710V4_) and strong SD sequence (eSD2), resulted in higher expression levels of heterologous protein and induced successful expression of the IH protein in *L. citreum* (Figure 2B). Therefore, we decided to use this strong expression system for subsequent conversion reactions in *L. citreum*.

### 2.2. Evaluation of Hydrolysis of Isoflavone Glycosides with Engineered L. citreum

**Figure 3:**
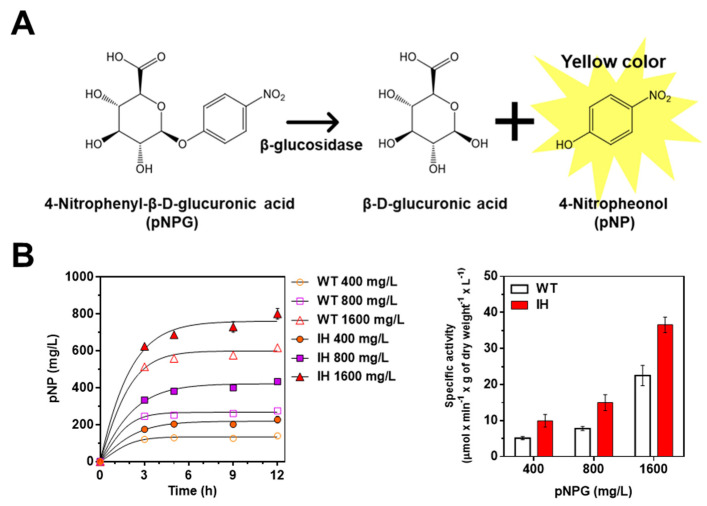
Identification of enzymatic activity of IH via in vivo β-glucosidase activity assay. (**A**) Schematic of reaction mechanism of β-glucosidase assay. (**B**) β-glucosidase activity of *L. citreum* wild type and *L. citreum* harboring pCB4270V4-IH in MRS medium supplemented with different concentrations of pNPG. The pNP production was measured at different culture times. Left, time profiles of pNP production; right, normalized specific activity of β-glucosidase activity of *L. citreum* wild type and *L. citreum* harboring pCB4270V4-IH at 12 h. WT represents *L. citreum* wild type and IH represents *L. citreum* harboring pCB4270V4-IH. Results are the mean of duplicate experiments, and error bars indicate standard deviations.

After we confirmed that the IH gene was properly expressed in *L. citreum* harboring pCB4270V4-IH, an in vivo β-glucosidase activity assay was performed to assess whether the synthesized IH was capable of being enzymatically active in *L. citreum*. The β-glucosidase activity was determined by bioconversion of 4-nitrophenyl-β-d-glucopyranoside (pNPG) into 4-nitrophenol (pNP) during bacterial cultivation (Figure 3A). The enzymatic product pNP was measured directly from the supernatant of the culture medium at an absorbance of 405 nm. Since MRS medium also displays absorbance at the same wavelength (405 nm), 0.1 M sodium carbonate was added to the supernatant to increase the molar extinction coefficient of pNP [32].

As expected, enzymatic activity of β-glucosidase was observed in both *L. citreum* wild type and *L. citreum* harboring pCB4270V4-IH (Figure 3B and Table 1) with various concentrations of pNPG (400, 800, and 1600 mg/L). As shown in Figure 3B, we found that *L. citreum* wild type also showed enzymatic activity for β-glucosidase, and it was assumed that the intrinsic β-glucosidases, due to their requirement in the metabolism of diverse carbon sources such as cellulose and lactose [33] in *L. citreum*, showed hydrolytic activity for pNPG regardless of their substrate specificity for isoflavone glycoside forms. Meanwhile, taking into consideration normalized specific activity of β-glucosidase, *L. citreum* harboring pCB4270V4-IH showed approximately two-fold higher activity than *L. citreum* wild type (Figure 3B and Table 1). This result indicated that the expressed IH in *L. citreum* harboring pCB4270V4-IH was biologically active in *L. citreum*.

**Figure 4:**
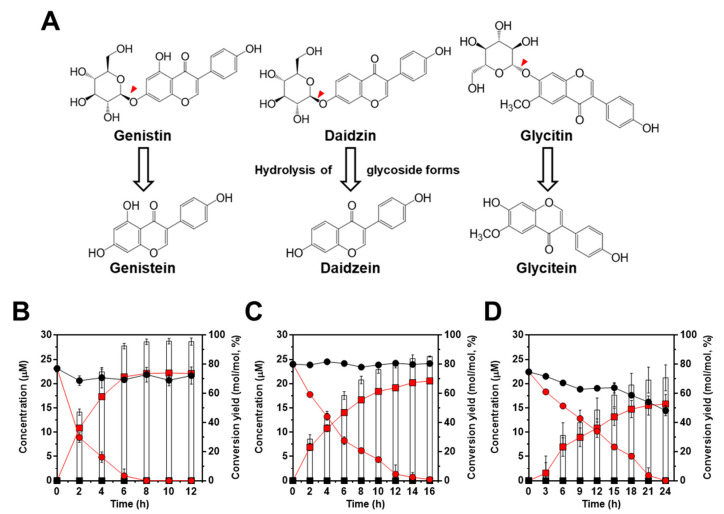
Bioconversion of isoflavones with standard chemicals by *L. citreum* wild type and *L. citreum* harboring pCB4270V4-IH. (**A**) Schematic diagram of bioconversion of isoflavone glycosides to aglycone forms. Red-color arrowheads indicate the hydrolysis site for IH. Bioconversion profiles of (**B**) 23.1 µM of genistin (100 mg/L) (**C**) 24 µM of daidzin (100 mg/L) and (**D**) 22.4 µM of glycitin (100 mg/L). Symbols: black circle, isoflavone glycoside forms in *L. citreum* wild type; black square, isoflavone aglycone forms in *L. citreum* wild type; red circle, isoflavone glycoside forms in *L. citreum* harboring pCB4270V4-IH; red square, isoflavone aglycone forms in *L. citreum* harboring pCB4270V4-IH, white bar, conversion yields of isoflavones in *L. citreum* harboring pCB4270V4-IH. Results are the mean of duplicate experiments, and error bars indicate standard deviations.

After we confirmed the enzymatic activity of IH using the β-glucosidase activity assay, bioconversion of isoflavone glycoside into aglycone forms was evaluated using three isoflavone glycosides (genistin, daidzin, and glycitin) which are major isoflavones in soybean (Figure 4A). As shown in Figure 4B–D, for genistin, daidzin, and glycitin, we found a high conversion yield of glycoside into aglycone forms in *L. citreum* harboring pCB4270V4-IH. First, contrary to the result of β-glucosidase activity observed in *L. citreum* wild type, bioconversion of isoflavone was not observed in wild type strain (Figure 4B–D). Therefore, it was confirmed that intrinsic β-glucosidases in *L. citreum* CB2567 did not have substrate specificity for isoflavone glycoside forms. Meanwhile, *L. citreum* harboring pCB4270V4-IH showed the highest activity for genistin among three examined substrates: 22.1 µM of genistein (aglycone form) was produced from 23.1 µM of genistin (100 mg/L) (glycoside form), with a conversion rate of 2.7 µM/h and a yield of 95% (0.95 mol/mol) at 8 h in *L. citreum* harboring pCB4270V4-IH (Figure 4B). Next, for 24 µM of daidzin (100 mg/L) (glycoside form), 20.5 µM of daidzein (aglycone form) was produced, with a conversion rate of 1.2 µM/h and a yield of 85% (0.85 mol/mol) at 16 h (Figure 4C). Finally, in the case of glycitin substrate, it was found that 15.8 µM of glycitein (aglycone form) was produced from 22.4 µM of glycitin (100 mg/L), with a conversion rate of 0.6 µM/h and a yield of 70% (0.70 mol/mol) at 24 h (Figure 4D). These results clearly confirmed that the engineered *L. citreum* had a highly efficient bioconversion property to convert all the isoflavone glycoside forms into aglycone forms.

### 2.3. Hydrolysis of Isoflavone Glycosides in Fermented Soymilk by Engineered L. citreum

**Figure 5:**
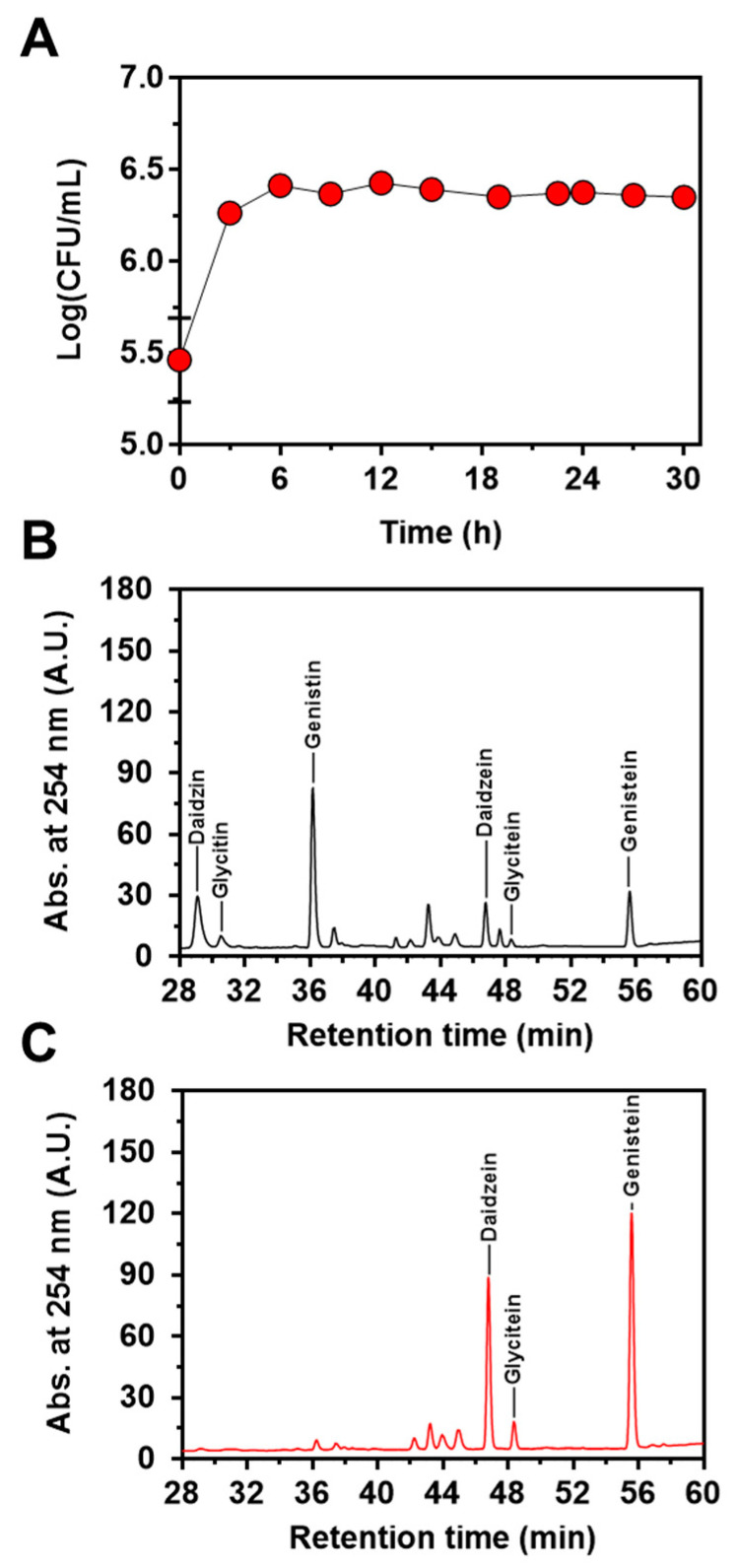
Soymilk fermentation of *L. citreum* harboring pCB4270V4-IH. (**A**) Growth profiles of *L. citreum* harboring pCB4270V4-IH. Results are the mean of duplicate experiments, and error bars indicate standard deviations. Chromatogram of HPLC analysis of isoflavones in soymilk during soymilk fermentation at (**B**) 0 h and (**C**) 30 h.

**Figure 6:**
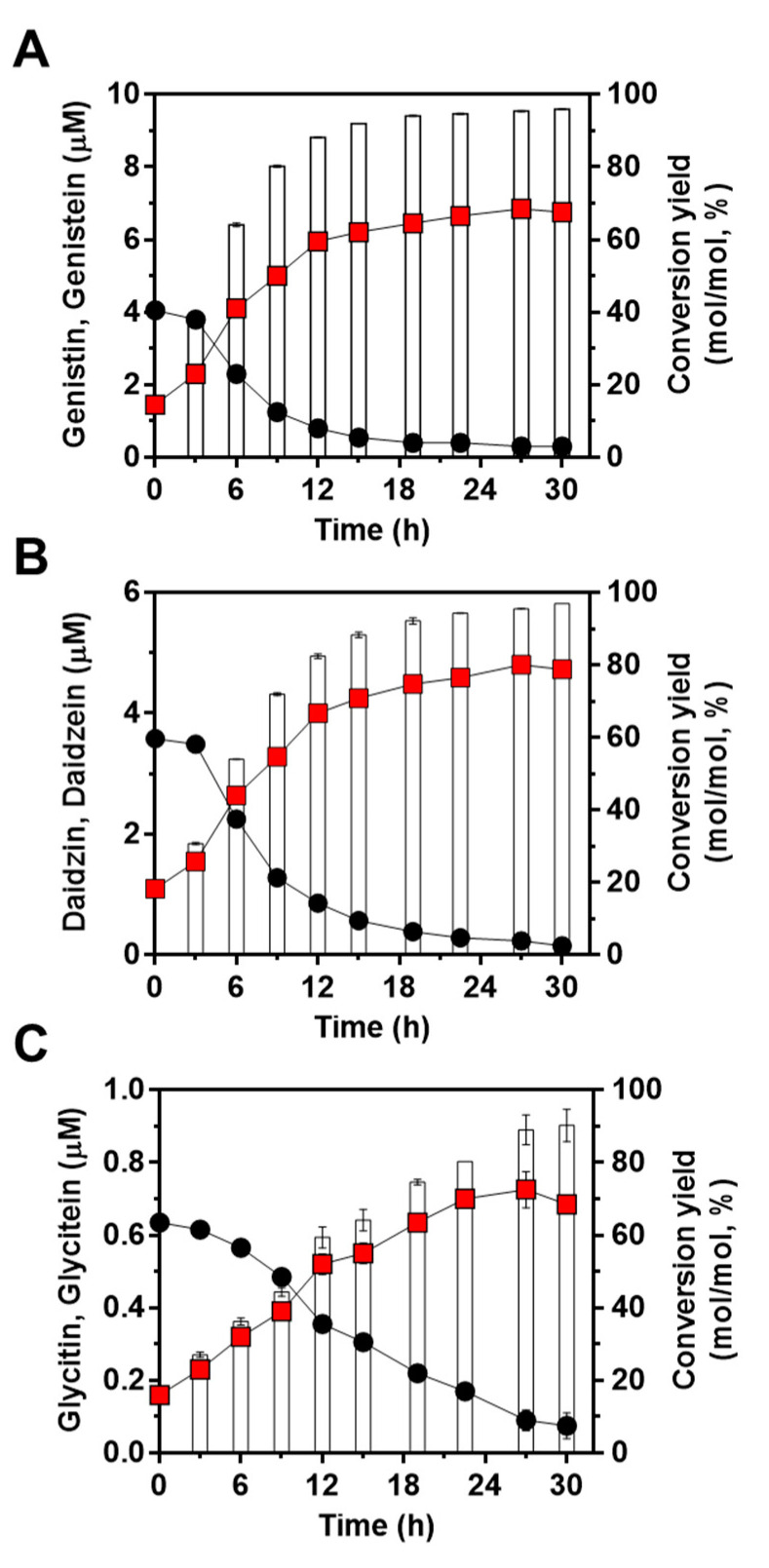
Bioconversion of isoflavones during soymilk fermentation by *L. citreum* harboring pCB4270V4-IH. (**A**) Production profiles of genistein. (**B**) Production profiles of daidzein. (**C**) Production profiles of glycitein. Symbols: black circle, isoflavone glycoside forms in soymilk; red square, isoflavone aglycone forms in soymilk; white bar, conversion yields of isoflavones in *L. citreum* harboring pCB4270V4-IH. Results are the mean of triplicate experiments, and error bars indicate standard deviations.

Next, to confirm the feasibility of the engineered strain of *L. citreum* to convert soy isoflavones during soymilk fermentation, cells were cultivated in 4% soymilk-containing media, and the concentrations of the three isoflavones (genistin, daidzin, and glycitin) and their aglycons (genistein, daidzein, and glycitein) were determined during 30 h of fermentation. As shown in Figure 5A, *L. citreum* harboring pCB4270V4-IH showed a constant growth rate until 6 h of fermentation and reached a stationary phase during the fermentation process. All the soy isoflavones, including genistin, daidzin, and glycitin, were successfully converted to aglycone forms by *L. citreum* harboring pCB4270V4-IH during soymilk fermentation (Figure 5B,C). *L. citreum* harboring pCB4270V4-IH gradually converted 4 µM genistin present in soymilk to 6.8 µM of genistein, with a conversion rate of 0.23 µM/h and a yield of 95% (0.95 mol/mol) (Figure 6A). Next, about 3.5 µM of daidzin present in soymilk was converted to 4.7 µM of daidzein, with a conversion rate of 0.16 µM/h and a yield of 96% (0.96 mol/mol) (Figure 6B). Finally, about 0.63 µM glycitin was converted to 0.68 µM of glycitein, with a conversion rate of 0.02 µM/h and a yield of 90% (0.90 mol/mol) (Figure 6C). All conversion data are summarized in Table 2. These results clearly confirmed that the engineered *L. citreum* had a highly efficient bioconversion property to convert all the isoflavone glycosides’ forms into aglycone forms, even during fermentation of soymilk.

With advances in synthetic biology and metabolic engineering during the last two decades, various synthetic moieties for gene expression have been developed, which offer innovative approaches for redesigning the existing biological systems in many bacterial hosts, including LABs, rendering them to be increasingly used as cell factories [34,35]. In this study, using a high copy number plasmid (pCB4270) and bicistronic expression design with synthetic parts [24,25,26], an optimized expression module for IH that mediates the conversion of isoflavone glycoside to aglycone forms was successfully constructed in *L. citreum*. To the best of our knowledge, this is the first report on the engineering of *L. citreum* to be used as a whole-cell biocatalyst for production of isoflavone aglycones, and our *L. citreum*-based novel platform has advantages over other bacterial hosts. To date, most of the studies on the hydrolyzation of isoflavone glycoconjugates in fermented soy products have focused on *Bifidobacterium* strains [36,37,38,39,40,41]. However, constraints regarding the cultivation in anaerobic conditions, lack of useful expression systems, and genome editing tools for *Bifidobacterium* strains limit their utilization in expanding the bioavailability of soy isoflavones. In addition, most intestinal bacteria including *Bifidobacterium* strains have metabolic pathways that degrade isoflavone aglycone forms into a variety of metabolites, such as dihydroisoflavones including dihydrogenistein, dihydrodaidzein, and dihydroglycitein [42,43,44], which cause a loss of active aglycone forms. In contrast, in *L. citreum,* no inherent metabolic pathway for the degradation of isoflavone aglycone forms has been reported. As shown here (Figure 5 and Figure 6), we could not observe the degradation of isoflavone aglycone during hydrolysis with our *L. citreum*-based whole cell system, so high conversion yields could be achieved without loss of aglycone. In addition, as shown here, various genetic tools are available in *L. citreum*, which will make this host more potential. It is proven that the engineered *L. citreum* can be a novel starter culture for soymilk fermentation while not only increasing the content of aglycones but also enhancing both acceptability and nutritional value of fermented soymilk, owing to its intrinsic properties for end products of carbohydrates. Recent scientific advancements clarified the relationship between the diet of soy isoflavones and human health. Particularly, their property for phytoestrogen with a similar structure to 17-β-estradiol allows them to decrease the risk of hormone-related diseases including breast and prostate tumors and even osteoporosis [2,3]. Furthermore, their antioxidant properties contribute to protecting neurodegenerative disease and photoaging effects by insulating ultraviolet (UV) radiation and chemicals [1,4]. In this regard, expectations for the utilization of soy isoflavones are drawing attention more and more, and we believe that our *L. citreum*-based whole-cell biocatalyst system will contribute to the development of an industrial platform for enhancing the bioavailability of soy isoflavones.

## 3. Materials and Methods

### 3.1. Bacterial Strains, Plasmids, and Growth Conditions

The bacterial strains and plasmids used in this study are listed in Table 3. *Escherichia coli* XL1-Blue was used for gene cloning and plasmid maintenance, and *L. citreum* CB2567 [45] was used as a major host for bioconversion of isoflavone glycosides. *E. coli* XL1-Blue was cultivated in Luria-Bertani medium (tryptone 10 g/L, yeast extract 5 g/L, and sodium chloride 10 g/L; BD, Franklin Lakes, NJ, USA) at 37 °C and 200 rpm. *L. citreum* was cultivated in MRS medium (proteose peptone no. 3 10 g/L, beef extract 10 g/L, yeast extract 5 g/L, dextrose 20 g/L, polysorbate 80 1 g/L, ammonium citrate 2 g/L, sodium acetate 5 g/L, magnesium sulfate 0.1 g/L, manganese sulfate 0.05 g/L, and dipotassium phosphate 2 g/L, purchased as premixed media from BD) at 30 °C with shaking at 200 rpm. Ampicillin (100 µg/mL) and chloramphenicol (Cm, 10 µg/mL) were used for the selection and cultivation of *E. coli* and *L. citreum*, respectively.

### 3.2. Plasmid Construction

For gene expression in *L. citreum*, pCB4270 [24], a high copy number plasmid derivative of pCB42 [46] from *L. citreum* CB2567, was used as the backbone plasmid. Polymerase chain reaction (PCR) was performed using a C1000^TM^ Thermal Cycler (Bio-Rad, Richmond, CA, USA) and PrimeSTAR HS Polymerase (Takara, Shiga, Japan). Oligonucleotides used for the PCR are listed in Table 4. To express IH, the native IH gene was synthesized by Macrogen (Daejeon, Korea) and amplified by PCR using primers F-IH and R-IH (Table 4). Next, the PCR product was cloned into pCB4270V4 [24] using the Gibson Assembly method employing the Gibson Assembly Master Mix (NEB, Ipswich, MA, USA), which yielded pCB4270V4-IH, in which the IH gene was constitutively expressed under the bicistronic expression design [25]. Subsequently, pCB4270V4-IH was transformed into *L. citreum* CB2567 by electroporation with a capacitance of 25 µF and voltage of 1.0 kV and 400 Ω using 0.1 cm cuvettes and a MicroPulser (Bio-Rad). Transformed cells were recovered using MRS agar plates containing 10 µg/mL chloramphenicol at 30 °C for 16 h.

### 3.3. Protein Preparation and Analysis

Following overnight cultivation of the recombinant *L. citreum*, the cells were transferred to 5 mL of fresh MRS medium in a 50 mL tube at 1:50 dilution and grown at 30 °C with gentle shaking at 200 rpm. To identify the protein expression level of the IH gene, after cultivating for 12 h, the cells were harvested by centrifugation at 13,000 rpm for 5 min at 4 °C and washed twice with phosphate-buffered saline (PBS, containing 135 mM NaCl, 2.7 mM KCl, 4.3 mM Na_2_HPO_4_, pH 7.2). Later, a total protein fraction of the cells was prepared by sonication (amplitude 20%, time 7 min, using “5 s on and 3 s off” mode). The samples were then heated at 100 °C for 5 min, and proteins were separated using 12% SDS-PAGE at 250 V for 30 min. The proteins were subsequently transferred to a polyvinylidene fluoride (PVDF) membrane using a Trans-Blot^®^ SD Semi-Dry Transfer Cell (Bio-Rad) for 1.5 h at 70 mA. Next, the membrane was incubated with 5% (*w*/*v*) skim milk to block non-specific binding of antibodies and then incubated with Monoclonal ANTI-HIS^®^-Peroxidase (HRP) antibody (Sigma-Aldrich, St. Louis, MO, USA) diluted 1:5000 in Tris-buffered saline containing 0.1% Tween (TBST; 20 mM Tris, 150 mM NaCl, and 0.1% Tween^®^ 20 detergent). After washing, his-tagged IH (52 kDa) was detected using Amersham ECL Prime Western Blotting Detection Reagent (GE Healthcare, Chicago, IL, USA). Images were captured using the ChemiDoc imaging system (Bio-Rad).

### 3.4. In Vivo β-Glucosidase Activity Assay

To analyze enzymatic activity of IH in *L. citreum*, an in vivo β-glucosidase activity assay was performed. β-glucosidase activity was determined using various concentrations of pNPG (Sigma-Aldrich) and pNP (Sigma-Aldrich). *L. citreum* wild-type cells and *L. citreum* cells harboring pCB4270V4-IH were inoculated into 5 mL of MRS (BD) medium and cultivated for 16 h at 30 °C with shaking at 200 rpm. Next, they were transferred to 50 mL of MRS medium in 250 mL baffled flasks at 1:100 dilution and grown at 30 °C with shaking at 200 rpm. Cell growth was analyzed by measuring optical density at 600 nm (OD600) using a spectrophotometer (Optizen POP; Mecasys, Daejeon, Korea). After the stationary phase was reached, 10 mL of the cultured cells was harvested by centrifugation at 13,000 rpm for 5 min and washed with PBS. The washed cells were resuspended in 10 mL fresh MRS medium containing various concentrations of pNPG. At each time point, 200 µL of the sample was loaded into 1.5 mL tubes and harvested by centrifugation at 13,000 rpm for 5 min. The supernatant (150 µL) containing pNP as a product was transferred to a 96-well plate (SPL Life Sciences, Pocheon, Korea), and 50 µL of 0.1 M sodium carbonate was added to each well. The pNP concentration was determined at 405 nm using a microplate reader (Tecan, Männedorf, Switzerland).

### 3.5. Whole-Cell Bioconversion in a Shake Flask

*L. citreum* cells harboring pCB4270V4-IH were inoculated into MRS (BD) medium. After overnight cultivation, the cells were transferred into a 250 mL baffled flask containing 50 mL of fresh MRS medium at 1:100 dilution and grown at 30 °C with shaking at 200 rpm for 24 h. Next, 10 mL of the cultured cells were harvested by centrifugation at 13,000 rpm for 5 min and washed twice with PBS buffer. The washed cells were resuspended in 10 mL of fresh MRS medium containing isoflavone glycoside forms (genistin, daidzin, and glycitin; Sigma-Aldrich). All the conversion reactions in the flasks were performed at 200 rpm in a shaking incubator at 30 °C. To measure the amount of isoflavone glycoside and aglycone forms, the cells were pelleted by centrifugation at 13,000 rpm for 5 min, and the supernatants thus obtained were filtered using a 0.22 µm syringe filter (Futecs, Daejeon, Korea). The filtered supernatants were diluted in 80% methanol and analyzed by HPLC.

### 3.6. Soymilk Fermentation

Soymilk (4%, *w*/*v*) was prepared by dissolving soybean flour (Sigma-Aldrich) in deionized water and autoclaving at 121 °C for 15 min. *L. citreum* cells harboring pCB4270V4-IH were inoculated into MRS (BD) medium. After the cells were cultivated for 16 h at 30 °C with shaking at 200 rpm at 30 °C, they were transferred to 250 mL baffled flasks containing 50 mL of MRS medium at 1:100 dilution and grown at 30 °C with shaking (200 rpm) for 24 h. Later, the cells were transferred to 50 mL of 4% soymilk at 5% (*v*/*v*) dilution and incubated at 30 °C with shaking (200 rpm) for 30 h. To analyze growth profiles of *L. citreum* harboring pCB4270V4-IH, 10 µL of the soymilk sample was diluted with PBS buffer and spread onto MRS agar plates with 10 µg/mL chloramphenicol at 30 °C for 24 h. The number of bacteria (CFU/mL) was calculated using the recovered colonies. Extraction of isoflavones from the fermented soymilk was performed in triplicate. Briefly, 1 mL aliquots of the sample were freeze-dried using a lyophilizer (ilShinBioBase, Daejeon, Korea). The lyophilized samples were dissolved in 80% methanol and heated to 60 °C for 2 h to extract the isoflavones. Next, the samples were centrifuged at 13,000 rpm for 5 min, and the supernatants thus obtained were loaded onto HPLC vial to measure the amount of isoflavone using HPLC.

### 3.7. HPLC Analysis

The HPLC system (Shimadzu, Kyoto, Japan) consisted of a pump (LC-20AD), autosampler (SIL-30AC), column oven (CTO-20 A), and refractive index detector (RID-10 A), and was equipped with a Zorbox Eclipse AAA column (150 × 4.6 mm, 3.5 microns; Agilent Technologies, PA, CA, USA). Samples were fractionated using a binary nonlinear gradient with mobile phase A (0.1% acetic acid in deionized water) and mobile phase B (0.1% acetic acid in acetonitrile). The column temperature was 35 °C, flow rate was 0.5 mL/min, and UV detection wavelength was 254 nm. The percentages of the mobile phases and the time periods were as follows: 95% mobile phase A for 4 min, 65% mobile phase A for 48 min, 15% mobile phase A for 54 min, 100% mobile phase B for 56 min, 100% mobile phase B for 66 min, 95% mobile phase A for 75 min, and 95% mobile phase A for 80 min.

## Figures and Tables

**Table 1:** Summary of in vivo β-glucosidase activity in *L. citreum* harboring pCB4270V4-IH.

Strain	pNPG (mg/L)	pNP ^a^ (mg/L)	Specific Activity ^a^ (µmol × min^−1^ × g of Dry Weight^−1^ × L^−1^)
*L. citreum* wild type	400	140.2 ± 2.8	5.1 ± 0.4
	800	275.5 ± 7.0	7.7 ± 0.5
	1600	617.3 ± 10.0	22.4 ± 2.8
*L. citreum* harboring pCB4270V4-IH	400	227.6 ± 17.4	9.9 ± 1.7
	800	443.4 ± 9.2	14.9 ± 2.2
	1600	800.6 ± 29.2	36.5 ± 2.1

^a^ Concentration of pNP and specific activity were calculated at 12 h. Data are summarized as mean ± standard deviation (*n* = 2).

**Table 2:** Summary of isoflavone aglycone forms production with soymilk fermentation.

Product	Concentration ^a^ (µM)	Conversion Rate ^a^ (µM/h)	Conversion Yield ^a^ (mol/mol, %)
Genistein	6.75 ± 0.07	0.23 ± 0.0	95.9 ± 0.1
Daidzein	4.72 ± 0.06	0.16 ± 0.0	96.9 ± 0.0
Glycitein	0.68 ± 0.02	0.02 ± 0.0	90.0 ± 4.4

^a^ Concentration, conversion rate, and conversion yield of the products were calculated for 30 h. Data are summarized as mean ± standard deviation (*n* = 3).

**Table 3:** List of strains and plasmids used in this study.

Strains/Plasmids	Description	Reference
Strains		
*E. coli* XL1-Blue	*recA1 endA1 gyrA96 thi-1 hsdR17 supE44 relA1lac [F’ proAB lacI^q^Z DM15 Tn10 (Tet^r^)]*	Stratagene ^a^
*L. citreum* CB2567	Wild type	[45]
Plasmids		
pCB4270	*L. citreum*-*E. coli* shuttle vector, P_710_, Amp^R^, Cm^R^	[26]
pCB4270V4	*L. citreum*-*E. coli* shuttle vector, P_710V4_, Amp^R^, Cm^R^	[24]
pCB4270V4-IH	pCB4720V4 derivative carrying IH encoding gene, P_710V4_, eSD2, IH, Amp^R^, Cm^R^	This study

^a^ Stratagene, La Jolla, CA, USA.

**Table 4:** Primers used in this study.

Primer Name	Sequence (5′ to 3′)
F-IH	GATGAAAGCAATTTTCGTACTGAAACATCTTAATCATGGAAGGGAGGGTTTTTAATGACGATGACGTTCCCGAA
R-IH	GCCTAGTGGTGGTGGTGGTGGTGCTTGGCGGAGTGCTCGGCG

## Data Availability

Not applicable.

## Abbreviations

LAB	Lactic acid bacteria
IH	Isoflavone hydrolase from *Bifidobacterium animalis* subsp. *lactis* SH5
